# Best practices for the manual curation of intrinsically disordered proteins in DisProt

**DOI:** 10.1093/database/baae009

**Published:** 2024-03-12

**Authors:** Federica Quaglia, Anastasia Chasapi, Maria Victoria Nugnes, Maria Cristina Aspromonte, Emanuela Leonardi, Damiano Piovesan, Silvio C E Tosatto

**Affiliations:** Institute of Biomembranes, Bioenergetics and Molecular Biotechnologies, National Research Council (CNR-IBIOM), Via Giovanni Amendola, 122/O, Bari 70126, Italy; Department of Biomedical Sciences, University of Padova, Via Ugo Bassi, 58/B, Padova 35131, Italy; Biological Computation & Process Laboratory, Chemical Process & Energy Resources Institute, Centre for Research & Technology Hellas, 6th km Harilaou - Thermis 57001 Thermi, Thessalonica 57001, Greece; Department of Biomedical Sciences, University of Padova, Via Ugo Bassi, 58/B, Padova 35131, Italy; Department of Biomedical Sciences, University of Padova, Via Ugo Bassi, 58/B, Padova 35131, Italy; Department of Biomedical Sciences, University of Padova, Via Ugo Bassi, 58/B, Padova 35131, Italy; Department of Biomedical Sciences, University of Padova, Via Ugo Bassi, 58/B, Padova 35131, Italy; Department of Biomedical Sciences, University of Padova, Via Ugo Bassi, 58/B, Padova 35131, Italy

## Abstract

The DisProt database is a resource containing manually curated data on experimentally validated intrinsically disordered proteins (IDPs) and intrinsically disordered regions (IDRs) from the literature. Developed in 2005, its primary goal was to collect structural and functional information into proteins that lack a fixed three-dimensional structure. Today, DisProt has evolved into a major repository that not only collects experimental data but also contributes to our understanding of the IDPs/IDRs roles in various biological processes, such as autophagy or the life cycle mechanisms in viruses or their involvement in diseases (such as cancer and neurodevelopmental disorders). DisProt offers detailed information on the structural states of IDPs/IDRs, including state transitions, interactions and their functions, all provided as curated annotations. One of the central activities of DisProt is the meticulous curation of experimental data from the literature. For this reason, to ensure that every expert and volunteer curator possesses the requisite knowledge for data evaluation, collection and integration, training courses and curation materials are available. However, biocuration guidelines concur on the importance of developing robust guidelines that not only provide critical information about data consistency but also ensure data acquisition.This guideline aims to provide both biocurators and external users with best practices for manually curating IDPs and IDRs in DisProt. It describes every step of the literature curation process and provides use cases of IDP curation within DisProt.

**Database URL**: https://disprot.org/

## Introduction

Intrinsically disordered proteins (IDPs) and intrinsically disordered regions (IDRs) are key players in a plethora of biological processes (BPs), wielding their unique structural flexibility to participate in vital cellular functions (1). Their lack of a stable three-dimensional (3D) structure under physiological conditions, challenging traditional structural paradigms (2, 3), prompts the necessity for specialized biocuration efforts. The DisProt database stands as a gold standard resource in this endeavour, collecting manually curated experimental data that describe the multifaceted roles of IDPs and IDRs (4). The landscape of IDPs spans critical domains such as viral processes, autophagy and disease pathways, including cancer. Their ability to rapidly adapt their conformation allows them to engage in multiple interactions, modulating cellular responses (5, 6). As their relevance becomes more evident, the need for precise, comprehensive and reliable biocuration gains increasing importance.

Biocuration, the curation of biological information, plays an instrumental role in capturing the several aspects that characterize IDPs and IDRs. The DisProt database has etched itself as a pivotal resource, gathering an expansive collection of over 2600 entries across diverse species and biological kingdoms. Expert biocurators sift through the scientific literature to gain insight into structural states, transitions, interactions and functions associated with IDPs and IDRs.

In this context, these guidelines aim to illuminate the best practices for DisProt biocurators but also extend its reach to external users and experimental scientists interested in submitting new evidence of intrinsic disorder in DisProt. The DisProt curation guidelines may also be an example to other manually curated resources on how to adopt qualitative practices for biocuration and make them available to current and potential curators. The qualitative choices described in these guidelines have been extensively discussed and developed within the ELIXIR Intrinsically Disordered Proteins (IDP) Community (7) (URL: https://elixir-europe.org/communities/intrinsically-disordered-proteins), whose goal is indeed to promote standardization and development of tools and resources for the IDP data dissemination. By dissecting the intricate steps of literature curation and offering real-world use cases, this guideline fosters a comprehensive understanding of the curation process and underscores the importance of DisProt in advancing the knowledge of IDP biology. The guideline spans the landscape of IDP and IDR curation, encompassing all aspects of curation processes, i.e. data prerequisites, structural ontologies, literature retrieval strategies, functional annotations and submission procedures. Moreover, by exemplifying the curation of specific well-known IDPs, such as ATG8-interacting protein 2 and RAF proto-oncogene serine/threonine-protein kinase, this guideline showcases the various aspects of IDP and IDR curation within the DisProt framework.

## Overview of the IDP/IDR manual curation process

**Figure 1.**
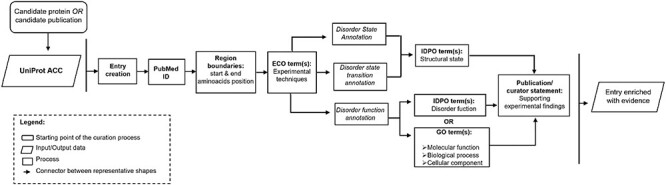
Workflow describing the important steps in the DisProt curation process.

The curation process in DisProt involves steps to annotate the disorder status of a protein, as well as allow enriching the entry with information about the transitional state or the disorder function. Figure 1 provides an overview of the curation process, encompassing the necessary information, to annotate the structural state and to enhance the entry with additional functional details.

In the prioritization process for manual curation in DisProt, proteins are selected based on their association with upcoming thematic datasets but also on the alignment with the research interests of curators, ensuring a focused and targeted approach to enhance the quality of the curated information.

The manual curation process in DisProt can start from one of the following two key methods: a candidate publication or a candidate protein. If curation begins with a candidate protein, a publication that characterizes the protein’s disordered nature must be identified using PubMed (8) or Europe PMC (9). If curation begins with a candidate publication, the proteins described in the publication must be identified. Regardless of the starting point, once a protein and a relevant publication have been chosen, the actual curation process is the same (as outlined in Figure 1). The beginning involves the identification of the UniProt ACC and the extraction of the intrinsic disorder-related information from the chosen publication.

As outlined in the workflow, it is crucial to emphasize that manual curation of a novel IDPs or IDRs (with at least 10 disordered residues) requires the following essential information:

- A ‘peer-reviewed article’ detailing the IDP/IDR, supported by reported scientific experiments.
- The identification of the protein’s ‘UniProt ACC’ mentioned in the publication paying attention to the ‘Organism’.
- At least one ‘experimental method’ defining the experimental setup used to generate the annotated information.
- The definition of the protein ‘region boundaries’ expressed as positions (start–end) in the corresponding UniProt entry.
- The annotation of the ‘intrinsic disorder structural state’ using IDPontology.
- A ‘publication statement’ consisting of sentences extracted from the publication, which provides information supporting the experimental findings and properly attributes the source of the presented information. Alternatively, a ‘curator statement’ contains sentences provided by the curator (often experts in a particular field/method), significantly describing the information extracted from the publication and offering insight into what viewers are observing.

It is also important to specify that if an entry associated with a specific UniProt ACC is already present in DisProt, a curator can add new evidence regarding its disordered state or evidence related to its functions. After defining the disorder state of a protein or region, the curator can add further evidence describing structural transitions or disorder-specific functions (Figure 1).

## Structuring data with the use of ontologies

In recent years, DisProt has been improved to facilitate structured curation with a controlled vocabulary using three ontologies:

- Intrinsically Disordered Proteins Ontology (IDPO), accessible at https://disprot.org/ontology, describes structural aspects, states, transitions of an IDP/IDR, as well as self-functions, and functions directly associated with their disordered state.
- Gene Ontology (GO) (10) (URL: http://geneontology.org/) is integrated to describe three key aspects within the biological domain related to IDP/IDR: molecular functions (MFs), BPs and cellular components (CCs).
- Evidence and Conclusion Ontology (ECO) (11) (URL: https://www.evidenceontology.org/) represents the experimental techniques used to assess the disordered structural state in a protein or related aspects.

Curators have the option to select ontological terms that best describe the information reported in the publication. For each piece of evidence that indicates the structural state, the disorder function and the applied method, curators should utilize the provided ontologies.

## Methods for retrieving IDP-related evidence

The extraction of disorder-related information can be one of the most significant challenges. Various strategies can be employed to retrieve information regarding the disorder status and functions of these proteins:

- Identify suitable publications that report experimental evidence of disorder state from ‘PubMed’ and ‘Europe PMC’. Curating IDPs/IDRs can be accomplished by constructing a ‘query’ using a combination of protein/gene names (or synonyms) and disorder-related keywords. Recommended keywords or effective detection of intrinsic disorder mention in a publication include terms such as ‘(intrinsic) disorder’, ‘unstructured’, ‘unfolded’, ‘flexible/flexibility’, ‘(high) mobility’, ‘missing residues’ and ‘electron density’. Authors sometimes list all the detected or missing electron density regions in their crystal structure but often without explicitly using the term ‘disorder’. It can also be helpful to search for terms like ‘visible’ or ‘missing’, which may indicate regions with the presence or absence of structure. If the terms mentioned earlier are not present in the publication, it may also be useful to search for terms that refer to experimental techniques frequently used to assess intrinsic disorder, such as ‘NMR’, ‘circular dichroism’, ‘SAXS’, etc.
- The second strategy involves consulting databases based on experimental methods or biological mechanisms closely associated with the disorder state. The databases mentioned in Table 1 are also cross-referenced in DisProt.

**Table 1.** The table shows a list of databases that can be consulted to retrieve information and references on experimental studies in proteins

Resource	Description	URL
Protein Data Bank in Europe (PDBe) (28)	X-ray crystallography, nuclear magnetic resonance (NMR)-related methods, and electron microscopy protein structures	https://www.ebi.ac.uk/pdbe
Biological Magnetic Resonance Bank (BMRB) (29)	Spectral and quantitative data derived from NMR spectroscopy	http://www.bmrb.wisc.edu
Small Angle Scattering Biological Data Bank (SASBDB) (30)	Small-angle x-ray scattering (SAXS) and small-angle neutron scattering experiments	https://www.sasbdb.org/
Electron Microscopy Data Bank (EMDB) (31)	Electron Microscopy Data Bank for electron cryo-microscopy, single-particle analysis, electron tomography, and electron crystallography	https://www.ebi.ac.uk/pdbe/emdb/
Protein Circular Dichroism Data Bank (PCDDB) (32)	Circular dichroism (CD) and synchrotron radiation CD (SRCD) spectral data and their associated experimental metadata	http://pcddb.cryst.bbk.ac.uk/home.php
PhaSePro (33)	Manually curated resource of proteins driving liquid–liquid phase separation (LLPS)	https://phasepro.elte.hu/
AmyPro	Validated amyloid precursor proteins and their amyloidogenic sequence regions	https://amypro.net/#/
Eukaryotic Linear Motif (ELM) (34)	Curated database of short linear motifs in eukaryotes	http://elm.eu.org/searchdb.html
UniProt (35)	Resource of protein sequence and functional information	https://www.uniprot.org/
MobiDB (36)	Database of protein disorder and mobility annotations	https://mobidb.bio.unipd.it/

Some of these resources pertain to techniques used in structural biology and are linked to scientific articles, while others, like MobiDB, serve as sources to extract information about the possible disorder state of a protein, which can be either predicted or curated. Indeed, in the last version of DisProt, another track specifically highlights the disordered regions derived from the missing residues of the PDB, as calculated by MobiDB (consensus trace)

## Finding a UniProt accession number for a protein

The peer-reviewed article used as the basis of the curation should ideally report the UniProt accession number of the protein described. If this information is available, the curator should use the specified UniProt accession. The use of UniProt as the reference for protein entry creation is a fundamental rule guiding DisProt curation process, ensuring consistency and reliability in data representation. By using UniProt, we can unequivocally create different protein entries that refer either to the canonical or isoform sequences. If this information is not available, the curator may encounter many exceptions:

- The authors use a different protein identifier (e.g. Ensembl ID, HUGO Gene Nomenclature Committee HGNC, etc.). In this case, the ID mapping service of UniProt (URL: https://www.uniprot.org/uploadlists/) to retrieve the corresponding UniProt accession is encouraged.
- The authors reference only the protein sequences. The curator should use that sequence as a query to search for a UniProt ACC using the built-in BLAST function. It is advisable to restrict the search criteria as much as possible (e.g. limiting to human proteins, vertebrates, bacteria, etc.).

The results of UniProt searches should be manually assessed, and the curator should use their judgement to select the most fitting protein. In the selection of the best match, the criteria described earlier in cross-checking (protein length, source organism, region boundaries, etc.) can be used. If the same sequence is present in UniProt under various accession numbers, priority should be given to SwissProt entries, if available. If the curator cannot find an available SwissProt entry, then the longest TrEMBL entry should be considered.

Discrepancies between UniProt and publication sequences can arise because of errors in the publication or modifications to the UniProt sequence after the publication date. In such cases, curators should compare the protein sequences, taking into account the provided sequence (if available from the authors), sequence length and region boundaries.

## Identifying and annotating the correct boundaries of a protein

Discrepancies may also occur for the sequence reported in the publication compared to the official UniProt one. This following curation example (DisProt entry DP02957, URL: https://disprot.org/DP02957) shows how curators should always check in UniProt the amino acid boundaries of the IDR described in the publication before annotating in DisProt. Indeed, in case of discrepancies between UniProt and the publication describing IDRs, the amino acid positions reported in UniProt prevail.

The authors of the paper ‘Monomeric solution structure of the prototypical “C” chemokine lymphotactin.’ published in *Biochemistry* (12) used nuclear magnetic resonance to analyse the IDRs of the human protein lymphotactin. The UniProt accession is P47992 (URL: https://www.uniprot.org/uniprotkb/P47992/entry). While it is not explicitly stated in the paper, the authors provide a GenBank accession (U23772) that can be mapped with the UniProt mapping service (URL: https://www.uniprot.org/id-mapping/) to retrieve the corresponding UniProt accession.

The authors of the publication provide several statements, extracted from different article sections, that should be used when annotating the IDRs of human lymphotactin in DisProt:

- Abstract: ‘Two regions are dynamically disordered as evidenced by 1H and 13C chemical shifts and {15N}-1H NOEs: Residues 1–9 of the amino terminus and Residues 69–93 of the C-terminal extension.’
- Results: ‘A steady decline is seen in the heteronuclear NOE values for the unstructured residues approaching the ends of the N- and C-termini with negative NOE values observed for residues near each terminus; these values are consistent with large-amplitude motions on the picosecond to nanosecond time scale and a complete lack of stable secondary or tertiary structure.’
- Results: ‘No long-range NOEs were observed for the 9 N-terminal and 26 C-terminal residues.’
- Results: ‘Residues 1–8 and 69–93 are highly disordered.’
- Discussion: ‘Residues comprising the unique C-terminal sequence of hLtn are entirely disordered in solution, but Residues 9–68 adopt the conserved fold observed for all other chemokines.’

**Figure 2.**
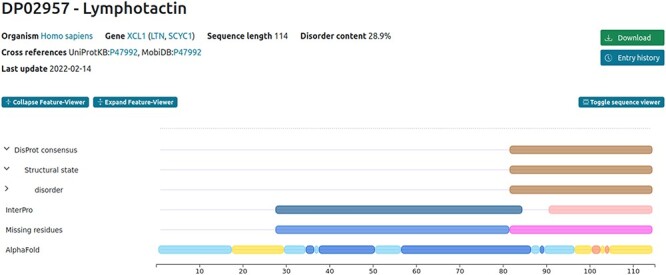
Example of the human lymphotactin protein available in DisProt.

In this example, two crucial factors need consideration when curating the disordered regions identified by the authors. Firstly, DisProt requires a minimum of 10 residues to annotate the structural state, rendering the initial region (Residues 1–8) too short for DisProt annotation. Secondly, the sequence used in the publication for defining IDRs does not align with the UniProt canonical sequence (P47992) of the protein under study. The experimental sequence in the publication corresponds to the mature secreted form of the protein, excluding the N-terminal signal sequence spanning the first 21 amino acids. To verify this, curators should refer to the ‘PTM/Processing’ section of the UniProt entry (P47992) to find evidence of the signal peptide (Regions 1–21) in human lymphotactin. This confirms that the studied sequence ranges from Residues 22 to 114, necessitating the annotation of boundaries in DisProt as 91–114 not 69–93 (Figure 2).

## Defining experiments and cross-references for an IDP/IDR

**Figure 3.**
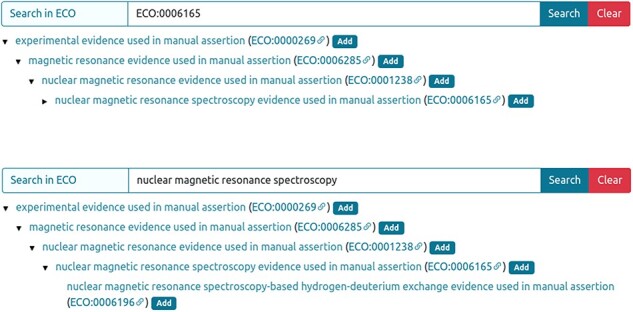
Entry curation page. Curator can retrieve the ECO technique using the ECO term (**A**) or the technique description (**B**) in the ‘Search in ECO’ bar, press the Search button and add the appropriate method.

The experimental technique used to analyse an IDP/IDR and its related disorder aspects can be annotated using ECO terms. When available, the most specific technique must be selected using the ECO term or the technique’s name (Figure 3).

Considering their elevated expertise, advanced or senior curators extend beyond the limitation of annotating purely experimental evidence. They are able to access restricted ECO terms, including ‘combinatorial experimental and curator inference evidence used in manual assertion (ECO:0007014)’ to provide an expert interpretation of IDP experiments. This ECO term is particularly valuable in situations where experimental outcomes leave uncertainties about the structural state of a region or protein. In such cases, senior curators may resort to supplementary resources such as structural predictors to gather more insights and elucidate the structural state of the region.

The curator is also encouraged to add cross-references to point to other databases, which can provide additional evidence regarding the disorder state or function. DisProt is currently cross-referenced to PDB, BMRB, PCDDB, SASBDB, EMDB, PhasePro, AmyPro and ELM. A list of source databases that can be cross-referenced in disorder-related publications is provided in Table 2.

**Table 2.** The table shows parameters and databases from which to retrieve information regarding the experimental components that the curator should annotate in the specific section of a DisProt entry

Sample component	Database
In-cell experiment	Cellosaurus (37)
Interacting antibody	ABCD (Antibodies Chemically Defined) (38)
Interacting lipid	CAS Registry Number (39)
	ChEBI (40)
	PubChem (41)
Interacting membrane	CAS Registry Number (39)
	ChEBI (40)
Interacting nucleic acid	ENA (European Nucleotide Archive)
	RNAcentral (42)
Interacting protein	CAS Registry Number (39)
	UniProt (35)
Interacting small molecule	CAS Registry Number (39)
	ChEBI (40)
	ChEMBL (43)
	PubChem (41)

**Figure 4.**
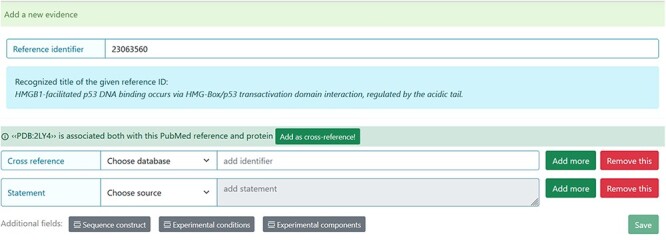
Entry curation page. Following the addition of the Reference identifier, the system automatically retrieves the PDB code related to the specified publication. Before adding it, the curator must verify that the information is correct.

The system automatically suggests the cross-reference after the PubMed Identifier (PMID) ID is added as evidence (Figure 4). For example, if the authors used X-ray for the structure detection and deposited the structure in the PDB database, the PDB code should be added. However, the curator should verify the information before accepting the suggestion.

## Identifying intrinsic disorder structural states and transitions

The following sections describe how to look for structural states and structural transitions pertaining to IDPs/IDRs in a publication.

### 8.1 Defining types of structural states

Four structural state terms are available in the IDP ontology. There are two high level terms to define the presence or the lack of structure (i and ii) and two subtypes of disorder to define a more specific structural state (a and b):

- Disorder: a non-compact state in which the protein lacks a stable 3D structure in isolation, covering both secondary structural elements and tertiary structure.
  - Pre-molten globule: a condensed but not compact state, with residual secondary structure, describing many native and non-native conformations in rapid equilibrium.
  - Molten globule: a compact state, with native secondary structure but lacking specific native tertiary structure.
- Order: a compact state with a stable 3D structure, in which most atoms have a fixed and stable position in relation to each other.

### 8.2 Defining types of structural transitions

The IDP ontology includes both transitions of IDPs and IDRs into a more ordered state or to a more disordered state as follows:

- Transitions to a more ordered state: disorder to pre-molten globule, disorder to molten globule, disorder to order, pre-molten globule to molten globule, pre-molten globule to order, molten globule to order.
- Transitions to a more disordered state: order to molten globule, order to pre-molten globule, order to disorder, molten globule to pre-molten globule, molten globule to disorder, pre-molten globule to disorder.

## Identifying functions associated with IDPs and IDRs

The following sections describe how to look for functions pertaining to IDPs/IDRs in a publication.

### Defining functions through GO terms

GO—the largest knowledgebase providing information on the functions of genes—can be used to describe functional aspects of an IDP or IDR. GO describes our knowledge of the biological domain with respect to three aspects:

- MF, molecular-level activities performed by gene products.
- BP, larger processes or ‘biological programs’ accomplished by multiple molecular activities.
- CC, cellular location where a gene product performs a function.

More information is available from the dedicated GO documentation page (URL: http://geneontology.org/docs/ontology-documentation/).

When annotating with GO, the most specific child term should be used. We recommend using AmiGO 2 (13) (URL: http://amigo.geneontology.org/amigo) or QuickGO (14) (URL: https://www.ebi.ac.uk/QuickGO/) in order to easily explore the available terms while curating the DisProt evidence.

Curators navigate to the ‘Function (GO)’ option in the ‘Aspect’ drop-down menu, prompting the display of a search box. Within the search box, curators can enter keywords to find relevant GO terms. For instance, entering ‘binding’ will yield options such as protein binding (GO:0005515), scaffold protein binding (GO:0097110), cytoskeletal protein binding (GO:0008092), and more. The specificity of the suggested GO terms increases with the accuracy of the search query.

### Defining disorder-derived functions

It is possible to annotate functions directly derived from the disordered state of the protein by using the IDP ontology. Detailed information about each term stored in IDP ontology is available in the Ontology page of DisProt (URL: https://disprot.org/ontology).

#### Entropic chain

Function directly arises from the lack of a stable structure. These entropic chain functions stem from the ability of the IDP to fluctuate between a large number of different conformational states. If known, the curator can be more specific about the type of entropic chain function by attaching terms under ‘entropic chain’ from the following: flexible C-terminal tail, flexible N-terminal tail and flexible linker/spacer.

#### Molecular recognition display site

The flexibility of a post-translational modification (PTM) site is usually required to allow it to effectively fit into the active site of the modifying enzyme; therefore, PTMs are usually associated with the presence of intrinsic disorder. Available terms under ‘molecular recognition display site’ include glycosylation display site, limited proteolysis display site and phosphorylation display site.

#### Self-regulatory activity

Protein interaction in *cis* that auto-regulates the protein function or its assembly, e.g. self-activation and self-inhibition.

## The MIADE guidelines: Minimum Information About a Disorder Experiment

The IDP Community has developed the Minimum Information About Disorder Experiments (MIADE) guidelines to unambiguously define an experimental setup used to study the structural aspects of IDPs or IDRs (15). As extensively described in the article, the MIADE guidelines provide recommendations for data producers on how to describe the results of their IDP-related experiments, for biocurators on how to annotate the experimental data in manually curated resources and for database developers on how to disseminate the data. In particular, MIADE increases the accuracy and accessibility of IDR annotations by providing information about experimental protocols, sample components or sequence properties that might affect the interpretation of the experimental results.

Using MIADE, it is possible to objectively examine and compare experimental evidence from other sources that follow the same standard. Curators can add experimental-related information in DisProt by clicking the ‘Additional fields’ buttons, which include ‘Sequence construct’, ‘Experimental conditions’ and ‘Experimental components’. For each annotation pertaining to this aspect, excerpts from the scientific article and ‘curators’ comments can be added to provide more clarity to the annotation.

### 10.1 Sequence construct information

**Figure 5.**
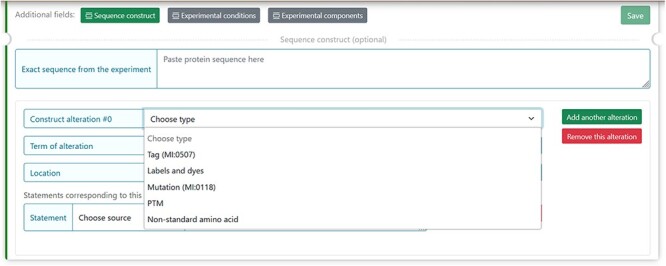
The figure shows the five selectable factors in DisProt to define any sequence differences reported in the experiment.

The MIADE implementation allows defining differences of the protein sequence described by the authors and UniProt protein sequence. Differences can arise from five factors that have been identified and described in the guidelines (Table 2 from (15)) and can alter the original sequence. In DisProt, it is possible to select one or more factors of the construct alterations and provide experimental details (Figure 5).

For each of the five factors, the construct alteration terms should be specified by choosing from the drop-down menu. Deviations from the canonical protein sequence should be described using appropriate ontologies:

Tag and labels by the format standard of molecular interaction data, PSI-MI (16) (URL: https://www.ebi.ac.uk/ols/ontologies/mi).

Mutations should be annotated using the Human Genome Variation Society (HGVS ) nomenclature for the description of protein sequence variants (17) (URL: https://varnomen.hgvs.org/).

PTMs and non-standard amino acids should be indicated using the controlled vocabulary for the protein–chemical modifications, PSI-MOD (18) (URL: https://www.ebi.ac.uk/ols/ontologies/mod).

**Figure 6.**
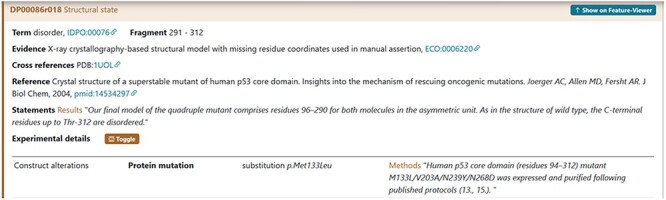
The figure shows the evidence of the Disordered Regions 291–312 experimentally detected. The construct used by the authors contains four substitutions M133L/V203A/N239Y/N268D reported with the HGVS nomenclature. The curator has also added a statement extracted from the article in the Methods section to support the evidence related to the construct alteration.

For instance, the experimental construct can differ from the canonical sequence by the presence of a mutation. For the cellular tumour antigen p53 (DisProt entry DP00086, URL: https://disprot.org/DP00086) (Figure 6), the sequence construct contains four non-synonymous substitutions, which nature should be specified in DisProt by selecting from the drop-down menu.

### Definition of the experimental conditions

**Figure 7.**
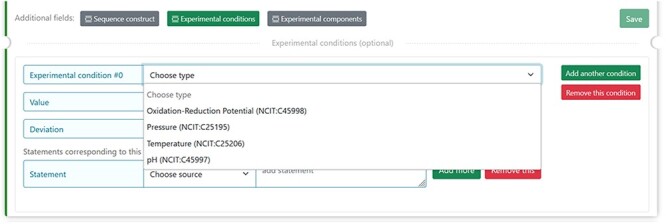
The figure shows the four selectable experimental parameters in DisProt to define any experimental setup for a sample in the study reported.

The experimental parameters of a given assessment can affect our comprehension of the biological significance of an experimental observation. Four parameter categories of the experimental setup for a sample, defined in the NCI Thesaurus OBO Edition controlled vocabulary (URL: https://ncit.nci.nih.gov/ncitbrowser/) (for details see Table 2 from (15)), should be specified in DisProt by choosing from the drop-down menu (Figure 7).

For each of the four properties absolute values (Units of Measurement Ontology) and deviations from the expected value (within normal range, increased, decreased, not specified or not relevant) can also be added.

**Figure 8.**
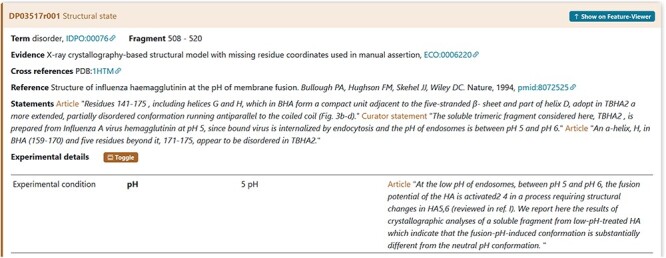
The figure shows the evidence of the Disordered Regions 508–520 experimentally detected. The soluble fragment is prepared at pH = 5 as mentioned in Experimental details. The curator has also added a statement extracted from the article to support the evidence related to the experimental conditions.

An example is the pH = 5 reported in a crystallographic analysis of a soluble fragment of hemagglutinin protein (DisProt entry DP03517, URL: https://disprot.org/DP03517) as reported in the details in Figure 8.

### Experimental components definition

Seven experimental sample components used by the authors during the characterization of an IDR should be specified in DisProt by choosing in the drop-down menu as shown in Figure 8.

The interacting partners that may affect the correct interpretation of the experiment have been defined in DisProt by the IDPO-controlled vocabulary.

For each of the experimental components, the curator should specify the database to cross-reference, based on the nature of the interaction partner (Table 2). However, the concentration and deviation can be added.

**Figure 9.**
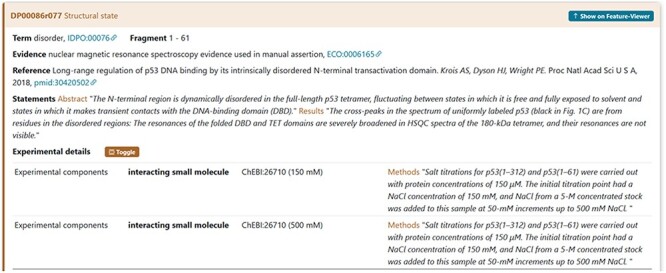
The figure shows the evidence of the Disordered Regions 1–61 experimentally detected. The sample studied by the authors contains the NaCl molecule. The curator has also added a statement extracted from the article in the Methods section to support the evidence related to the interacting partners.

An example is the interacting proteins used by the authors during the characterization of the IDR in cellular tumour antigen p53 (DisProt entry DP00086, URL: https://disprot.org/DP00086). The small interacting molecule with ChEBI ID: 26710 is sodium chloride (NaCl) as specified also in the statement by curators (Figure 9).

For further details and information about disorder-related experiments codified through MIADE in DisProt, we recommend curators to refer to the MIADE guidelines from Meszaros *et al*. (15).

## Thematic datasets

Since December 2020, DisProt has offered thematic datasets that are relevant to specific BPs or organisms (4, 19). The construction of these datasets relies on collaborations established among experts in the respective fields. Consequently, curators have the option to focus their curation efforts on proteins that are linked to a thematic dataset and contribute to their enrichment. They also have the opportunity to propose the creation of new thematic datasets.

The protein selection provided to curators for dataset construction or enrichment comes from reliable data sources, including curated databases specializing in particular topics. For instance, ‘Cancer-related proteins’ dataset has been constructed with proteins included in COSMIC (20). Similarly, for the ‘NDDs-related proteins’ dataset, proteins associated with neurodevelopmental disorders (NDDs) were selected from resources such as Simons Foundation Autism Research Initiative (SFARI) (21) and SysNDD (22).

**Figure 10.**
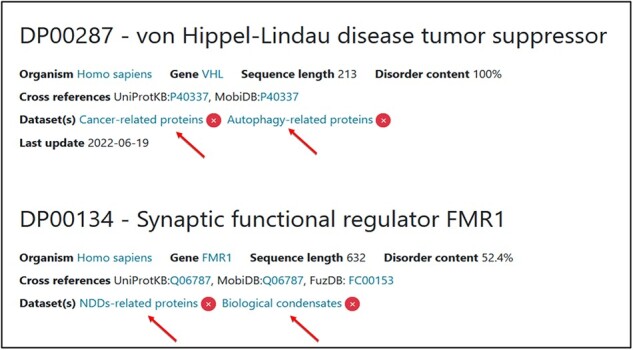
Two examples of proteins associated with two thematic datasets. Two tags have been added for each one of them.

Regarding the practical aspect, during the annotation process, the curator should also add a specific tag to the protein if it belongs to an available dataset (Figure 10).

### IDP literature curation use cases

**Figure 11.**
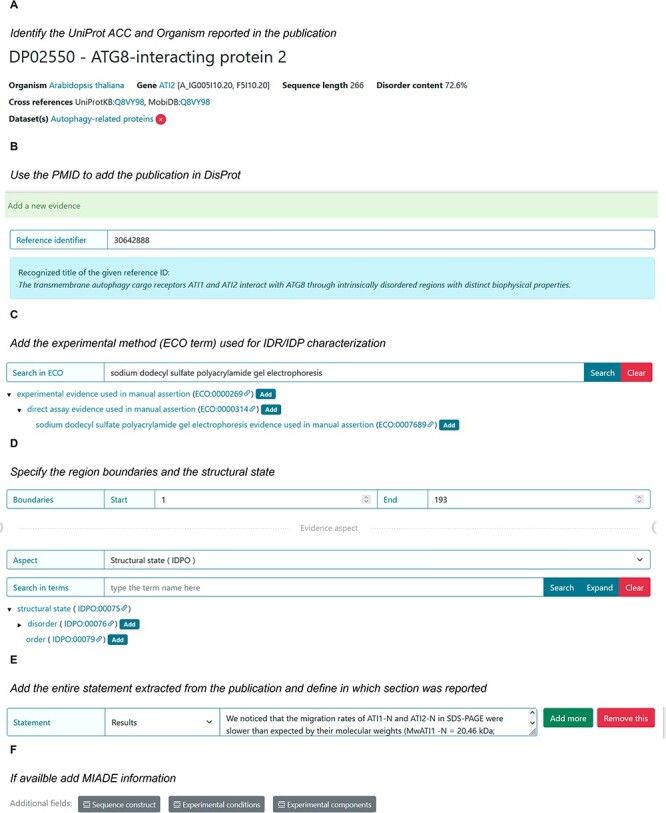
DisProt representation of the steps performed during the manual curation of ATG8-interacting protein. (**A**) A UniProt accession number should be used for the entry creation. (**B**) Include the PMID to cite the publication as the source of information. The title is automatically retrieved. (**C**) The method employed for the assessment should be chosen from the list of available ECO terms. (**D**) In accordance with the UniProt sequence, the curator should report the ‘start’ and ‘end’ positions (1–193) of the IDR. From the drop-down menu choose the structural state (IDPO) and select the specific term. (**E**) As support of the evidence and annotation, curators are required to add statements extracted from the Results of the publication. Curators should copy and paste the original sentences as they appear in the article. (**F**) Additional information corresponding to sequence construct, experimental conditions and /or experimental components can be added if suitable.

The following sections provide guidance on using data available within scientific articles to create an entry or new evidence for curating disordered proteins and regions in DisProt. Figure 11 shows an overview of the specific steps for adding information about an IDP/IDR for the ATG8-interacting protein 2 (ATI2) in DisProt.

### ATG8-interacting protein 2

To elucidate the disorder status and functional role of the N-terminus in the ATI2 protein, the authors of the article entitled ‘The transmembrane autophagy cargo receptors ATI1 and ATI2 interact with ATG8 through intrinsically disordered regions with distinct biophysical properties’ published in *The Biochemical Journal* (23), conducted various experiments and engaged in a comprehensive discussion regarding the IDRs and their significance within the ATI2 protein. The corresponding protein entry is already annotated in DisProt (DisProt entry DP02550, URL: https://disprot.org/DP02550).

#### Finding the UniProt accession number for ATI2 protein

The first step in curating the referenced publication is to identify the UniProt ACC for one of the two proteins mentioned in the paper, ATI2, paying attention to the organism considered: *Arabidopsis thaliana*. The authors refer to the protein by its official symbol name, ATI2, and the alternative name, At4G00355, corresponding to the Q8VY98 as UniProt ACC.

#### Identifying intrinsic disorder structural state of ATI2

The disorder status assessment of the ATI2 N-terminus is performed by five experiments (Table 3). The IDPO ontology available in DisProt should be used to indicate the nature of the N-terminal region of ATI2 protein with the specific term IDPO:00076: ‘disorder’, according to what is stated in the publication. In this particular case, the authors are very clear in defining the region of the ATI2 protein as disordered, both in the title and in the text. Hence, the curator ought to choose excerpts from the publication that unequivocally endorse and elucidate the disorder of the region, incorporating them as unmodified statements through the process of copying and pasting.

**Table 3.** The table contains data extracted from the publication (23) used for characterizing the ATI2 protein’s disordered state

Data to be annotated for the structural state	Description
Scientific publication	‘The transmembrane autophagy cargo receptors ATI1 and ATI2 interact with ATG8 through intrinsically disordered regions with distinct biophysical properties’ (23)
Organism	*A. thaliana*
Protein	ATG8-interacting protein 2 (ATI2)
UniProt ACC	Q8VY98
Region boundaries	1–193
Experimental methods for the IDR assessment	SDS-PAGE (Sodium Dodecyl-Sulfate Polyacrylamide Gel Electrophoresis), size-exclusion chromatography, far-UV CD, NMR spectroscopy and temperature-induced protein unfolding
Aspect of the annotation and term	Disorder
Publication statement	‘We noticed that the migration rates of ATI1-N and ATI2-N in SDS-PAGE were slower than expected by their molecular weights (MwATI1-N = 20.46 kDa; MwATI2-N = 21.02 kDa) (Figure 3A). This simple observation represents a first indication that ATI1-N and ATI2-N may be intrinsically disordered, because IDRs are typically depleted in hydrophobic residues, and, consequently, tend to bind less SDS, explaining their abnormally slow mobility in SDS-PAGE (21)’. (SDS-PAGE)

The region flexibility (1–193) was verified by five different techniques, all of which has been included in DisProt as five separate pieces of evidence, associated with each experimental method and supported by a corresponding statement from the publication. One statement as an example was reported in this table.

In this case, there will be five distinct pieces of evidence for the disorder status of the region ranging from 1 to 193 residues, each of them supported by different methods and supported by the corresponding statement from the publication (Table 3).

#### Defining the IDR functions of the ATI2 AIM motif through GO terms

By using two different methods (Table 4), the authors have further identified and characterized the presence of an ATG8-interacting motif (AIM) located at Positions 14–17 (residues WEVV) of ATI2, within the IDR (1–193). This feature should be annotated as a function employing the GO term ‘Protein binding’ (GO:0005515) where the binding partner, the autophagy-related protein 8f, should also be specified by its UniProt ACC (Q8VYK7).

**Table 4.** The table contains all data extracted from the publication (23) useful for characterizing the disorder function of ATI2 protein

Data to be annotated for the disorder function	Description
Scientific publication	‘The transmembrane autophagy cargo receptors ATI1 and ATI2 interact with ATG8 through intrinsically disordered regions with distinct biophysical properties’ (23)
Protein	ATI2
Organism	*A. thaliana*
UniProt ACC	Q8VY98
Experimental methods for IDR functional assessment	Heat treatment, yeast two-hybrid assays, NMR spectroscopy
Region boundaries	13–18
Curator statement	‘Region including the N-terminal AIM motif (WEVV) at position 14-17’.
MIADE	Experimental details: (i) Construct alterations (ii) Substitution: *p.Trp14Ala, p.Val17Ala*

Two different functions: protein binding and selective autophagy were demonstrated by the authors and supported in DisProt by corresponding statement and cross-reference. One statement as an example was reported in this table.

Additionally, this region is implicated in selective autophagy, as demonstrated by two techniques (Table 4). This functional attribute can also be annotated using the GO term for ‘selective autophagy’ (GO:0061912).

‘Molecular function—protein binding’, GO:0005515.

Biological process—selective autophagy, GO:0061912.

Note that in the DisProt annotations related to these features, the boundaries (13–18) differ from those reported in the publication and in the UniProt canonical isoform. This divergence results from functional annotation requirements for which a ‘minimum of 5 residues’ are necessary for the annotation in DisProt. Consequently, the curator should extend the boundaries by one amino acid.

### RAF proto-oncogene serine/threonine-protein kinase (RAF1)

The following example describes a RAF1 protein for which information about disordered state is derived from two publications ‘Synergistic binding of the phosphorylated S233- and S259-binding sites of C-RAF to one 14-3-3ζ dimer (24)’ published in *Journal of Molecular Biology* and ‘Stabilization of physical RAF/14-3-3 interaction by cotylenin A as treatment strategy for RAS mutant cancers (25)’ published in *Chem Biol*. The corresponding protein entry is already annotated in DisProt (DisProt entry DP00171, URL: https://disprot.org/DP00171).

**Figure 12.**
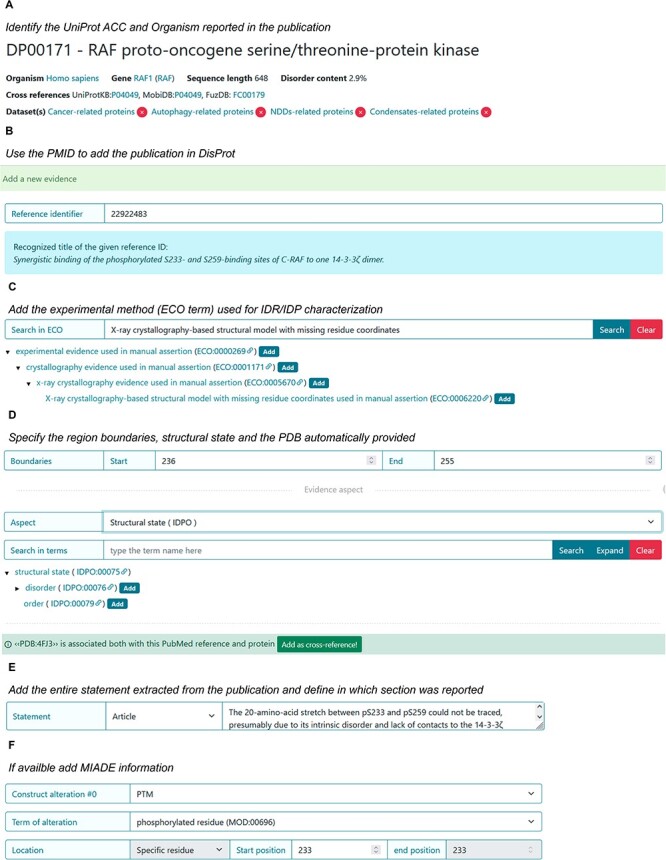
DisProt representation of the steps performed during the manual curation of RAF proto-oncogene serine/threonine-protein kinase. (**A**) UniProt accession number should be used for the entry creation. (**B**) Include the PMID to cite the publication as the source of information. The title is automatically retrieved. (**C**) The method employed for the assessment should be chosen from the list of available ECO terms. (**D**) In accordance with the UniProt sequence, the curator should report the ‘start’ and ‘end’ positions (236–255) of the IDR. From the drop-down menu, choose the structural state (IDPO) and select the specific term. Add the PDB as cross-reference automatically provided by the system. (**E**) As support of the evidence and annotation, curators are required to add statements extracted from the publication. Curators should copy and paste the original sentences as they appear in the article. (**F**) Additional information corresponding to PTM under sequence construct can be added.

The specific sections will be informative for curating the disordered state of a phosphorylated peptide when it is in complex with a protein partner and how to support the disordered state (e.g. Statement) and experimental conditions (MIADE) (Figure 12). It should be noted that in both publications, the structure of the di-phosphorylated RAF1 peptide in complex with the 14-3-3ζ shows an intrinsically disordered about 20 amino acids region.

#### Finding a UniProt accession number for RAF1 protein

The UniProt accession number was obtained by searching UniProt using the protein name, RAF1, and the organism of origin, *Homo sapiens*, as stated by the authors in the publication. The amino acid sequence of the UniProt entry P04049 (URL: https://www.uniprot.org/uniprotkb/P04049/entry) was compared to the amino acid sequence of the synthetic RAF1 construct used in the publication: ‘QHRYSTPHAFTFNTSSPSSEGSLSQRQRSTSTPNVH’ (shown in Figure 1) to ensure its identity.

#### Defining the experiments used to characterize RAF1

To determine the structural basis of the interaction between RAF1 and the 14-3-3ζ protein, the authors performed X-ray diffraction analysis of the crystallized complex. Even though the ECO term ‘X-ray crystallography evidence used in manual assertion’ (ECO:0005670) is acceptable to describe the technique, the more specific child term ‘X-ray crystallography-based structural model with missing residue coordinates used in manual assertion’ (ECO:0006220) is more suitable and should be selected to describe the experimental procedure in which the authors based their observations about RAF1 structural state. After adding both PMIDs, the system will automatically provide the PDBs code as cross-references. In this case, since the inserted technique is X-ray and the amino acid residues mentioned by the authors are missing, PDB:4FJ3 and PDB:4IHL can be added by the curators.

#### Identifying intrinsic disorder structural state of RAF1

Molzan *et al*. (25) co-crystallized the 14-3-3ζ protein with a synthetic di-phosphorylated peptide of 36 amino acids that corresponds to the 229–264 residues of RAF1. While it was possible to crystallize 14 residues of RAF1, associated with the 14-3-3ζ interacting region, it was not possible to trace a 20-amino‐acid stretch between the phosphorylated sites (Ser 233 and Ser 259).

The lack of electron density in the Regions 236–255 indicates significant flexibility, signifying an IDR, as defined by the IDPontology term ‘*dis*order’ (IDPO:00076). In the work of 2013 by Molzan *et al*. (25), the 14-3-3ζ protein was crystallized in the presence of the same synthetic peptide (RAF1) and the natural product cotylenin A. In this case, the disordered stretch was shorter than the previous one, 238–254, (Table 5) and the authors did not explicitly restate its disordered nature. Nevertheless, based on the observed missing electron density in the PDB file, the curator can independently confirm the disorder status of this region (Figure 6).

**Table 5.** The region flexibility (238–254) was verified by X-ray technique, included in DisProt and supported by a corresponding statement from the publication

Data to be annotated for the structural state	Description	Description
Scientific publication	‘Synergistic binding of the phosphorylated S233- and S259-binding sites of C-RAF to one 14-3-3ζ dimer’	‘Stabilization of physical RAF/14-3-3 interaction by cotylenin A as treatment strategy for RAS mutant cancers’
Organism	*H. sapiens*	*H. sapiens*
Protein	RAF proto-oncogene serine/threonine-protein kinase	RAF proto-oncogene serine/threonine-protein kinase
UniProt ACC	P04049	P04049
Experimental methods for IDR assessment	X-ray crystallography	X-ray crystallography
Region boundaries	236–255	238–254
Statement	From article	By curator
MIADE	Construct alterations Experimental components	Construct alterations Experimental components

One statement as an example was reported in this table.

#### Defining MIADE specifications

As stated previously, modifications of the amino acid sequence or the presence of molecular partners could affect the result of a given experiment and should be taken into consideration for its correct interpretation.

**Figure 13.**
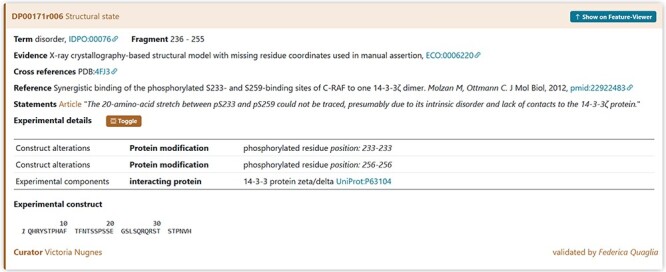
A piece of evidence of the structural state for the RAF1 IDR (236–255).

The annotation regarding the X-ray evidence of disorder region in RAF1 should also report the presence of the interacting protein 14-3-3ζ (UniProt ACC P63104) and the presence of the PTM modifications of the Serine Residues 233 and 256 (Figure 13). This information can be found in the PDB-deposited structure and in the method section of the publication.

## Training courses: future prospective

The training activity in DisProt is the foundation for the curation, implementation and expansion of the database. Researchers interested in joining the DisProt curation team can reach out at disprot@ngp-net.bio.unipd.it. Every curator, before receiving their account for curation in DisProt, should complete a course available on the ELIXIR eLearning platform (URL: https://elixir.mf.uni-lj.si/enrol/index.php?id=91). The course, currently available in English and Spanish, provides curators with most relevant information to start their biocuration activity.

Other curation materials are available as webinars and curation manuals. In particular, in ELIXIR TeSS (Training eSupport System), it is possible to find a beginner and intermediate level course describing the DisProt resource (URL: https://tess.elixir-europe.org/search?q=disprot). A team of experts will be engaged to video-record training sessions focused on experimental methods, making them accessible to anyone wishing to deepen their knowledge and interpretation of disorder-related data experimentally studied in the literature.

## Recognition and accreditation in DisProt

One of the most important policies of DisProt is to consistently reward the effort and meticulous work of expert and volunteer curators. In this context, DisProt was one of the first databases to be integrated into APICURON, a platform developed with the purpose of accrediting the work of biocurators based on the concept of gamification (URL: https://apicuron.org/) (26). The recognition of a DisProt biocurator’s activity takes into account the effort, accuracy and data quality added to the DisProt database. The terms and scores that accredit the curator’s activity are based on the importance and the purpose of encouraging further exploration of that data in DisProt.

## Submitting an annotation of intrinsic disorder to DisProt

**Figure 14.**
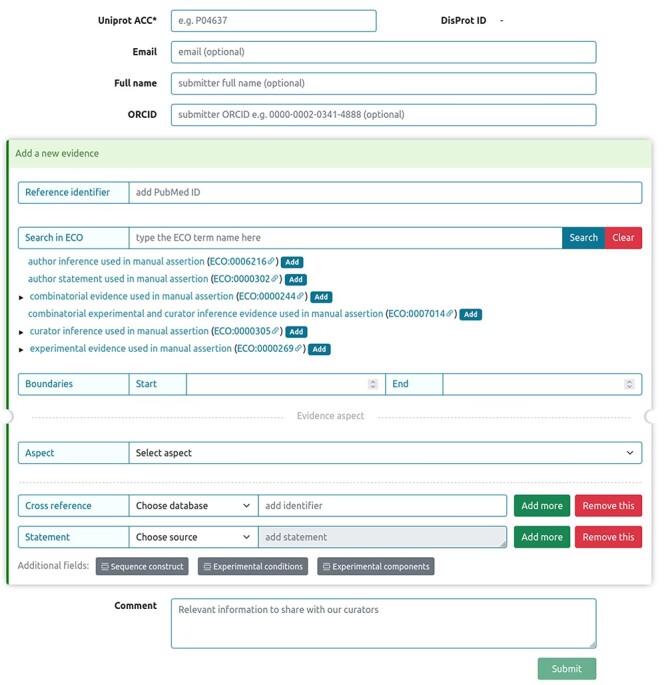
DisProt submission form for external users.

Sharing novel insights by external users, as well as experimentalists working on IDPs and IDRs into the DisProt database, was streamlined through a dedicated submission form, ensuring the integration of manually curated literature findings. Figure 14 shows the curation process available in DisProt submission form, accessible through the DisProt website (URL: https://disprot.org/biocuration). External users can find all necessary fields for accurate annotation. Initiating the process, users provide the ‘UniProt ACC’ for precise cross-referencing, and if available, the DisProt identifier in order to enhance the linkage. Essential contact information, including ‘Email’, ‘Full name’ and ‘ORCID’, guarantees proper attribution to the author of the submission. Contributors are required to add, for each new submitted evidence, the PubMed reference identifier of the peer-reviewed scientific publication describing the IDP/IDR, as well as the characterized region, the experimental technique and the disorder aspect described. Contributors also have the chance to fill out additional fields, such as ‘Cross reference’ and ‘Statement’, to contextualize the annotation. The submission process accommodates for additional information in the ‘Comment’ section, fostering clarity.

## Conclusions

IDPs and IDRs represent essential components of the proteome, playing diverse and pivotal roles across BPs and functions. Biocuration ensures an accurate and standardized representation of the inherent complexity of IDP and IDR properties, functions and interactions, thus facilitating a comprehensive understanding of their different contributions to cellular dynamics.

The biocuration of these proteins from peer-reviewed literature forms the basis of knowledge enrichment within the DisProt database.

The process of curation is well defined and follows a structured approach to help curators carefully extract relevant information from scientific publications. This includes precise delineation of protein boundaries, thorough documentation of experimental methods and meticulous recording of disorder-related details.

The DisProt database integrates ontologies like the IDPO, GO and the ECO to provide structured data annotations. These ontologies bring consistency to the classification of IDP/IDR attributes, ranging from structural states and transitions to MFs and BPs, resulting in coherent cross-referencing and interpretation.

Moreover, incorporating the principles of the MIADE guidelines, we elevate our curation practices. MIADE guidelines serve as the foundation upon which we build our data representation. They ensure that every piece of relevant information is captured and presented with the utmost precision, resulting in comprehensive and trustworthy data sets.

This guideline, designed for both biocurators and external users, provides a step-by-step guide for the systematic and thorough curation of IDPs and IDRs within the DisProt framework.

By addressing every aspect of the curation process and by providing practical examples of well-known IDPs, this guideline allows curators and users to explore the rigorous curation best practices that ensure the maintenance of DisProt’s high standards of data accuracy and reliability.

In addition, users and curators interested in the DisProt database and in the interpretation of its manually curated annotations can refer to a specific protocol providing a detailed description on the use of DisProt (27).

These guidelines serve as a foundation for ensuring the reproducibility of data curation within DisProt, by explaining in detail each step of the literature curation process and providing use cases. The aim of these guidelines is to help current and prospective biocurators understand how to perform curation in our database by following reproducible best practices.

## Data Availability

The data underlying this article are available in DisProt, at https://disprot.org/.
